# Screen time and depression risk: A meta-analysis of cohort studies

**DOI:** 10.3389/fpsyt.2022.1058572

**Published:** 2022-12-22

**Authors:** Liqing Li, Qi Zhang, Liyong Zhu, Guohua Zeng, Hongwei Huang, Jian Zhuge, Xiaorui Kuang, Sule Yang, Di Yang, Zhensheng Chen, Yong Gan, Zuxun Lu, Chunmei Wu

**Affiliations:** ^1^Research Center of Health Policy and Innovation, Jiangxi Science and Technology Normal University, Nanchang, Jiangxi, China; ^2^School of Public Health, Tongji Medical College, Huazhong University of Science and Technology, Wuhan, Hubei, China; ^3^School of Economics and Management, Jiangxi University of Science and Technology, Ganzhou, Jiangxi, China; ^4^Department of Health Management Medicine, The Second Affiliated Hospital of Nanchang University, Nanchang, China; ^5^School of Public Health and Health Management, Gannan Medical University, Ganzhou, Jiangxi, China

**Keywords:** screen time, depression, meta-analysis, mental health, epidemiology

## Abstract

**Background:**

The impact of screen time on mental health, including depression, has attracted increasing attention from not only children and adolescents but also the elderly. Thus, we conducted a meta-analysis of cohort studies to evaluate the association between screen time and depression risk.

**Methods:**

The PubMed, Embase, Web of Science, and China National Knowledge Infrastructure databases were searched for cohort studies up to May 2022, and the reference lists of the included studies were also retrieved. A random-effect model was used to estimate the combined effect size. Heterogeneity was assessed with the *I*^2^ statistic. Potential publication bias was evaluated using a funnel plot and Begg’s and Egger’s tests.

**Results:**

The final analysis included 18 cohort studies with a combined total of 241,398 participants. The pooled risk ratio (RR) was 1.10 (95% confidence interval: 1.05–1.14), with significant heterogeneity (*I*^2^ = 82.7%, *P* < 0.001). The results of subgroup analyses showed that the pooled RRs varied according to geographic locations, gender, age group, screen time in the control group, depression at the baseline, and whether the study was conducted during the COVID-19 pandemic. No obvious evidence of publication bias was found.

**Conclusion:**

This study indicates that screen time is a predictor of depressive symptoms. The effects of screen time on depression risk may vary based on the participant’s age, gender, location, and screen time duration. The findings could have important implications for the prevention of depression.

## 1 Background

Depression is a common and disabling psychiatric condition worldwide (1). According to estimates of the Global Burden of Diseases (GBD), injuries, risk factors study, depressive disorders accounted for 170.8 million cases in 1990 and 279.6 million cases in 2019, representing a 63.7% increase in prevalence (2). Depressive etiology is complex and results from interactions between biological vulnerabilities and environmental factors (3). According to the GBD (2), depression has been among the three top causes of non-fatal health losses for the past 30 years. Furthermore, research shows that depression is expected to be the leading global cause of disability-adjusted life year by 2030 (4). Depressive and anxiety disorders account for between one-quarter and one-third of all primary healthcare visits worldwide, and the World Health Organization (WHO) has estimated that neuropsychiatric disorders account for 1.2 million deaths annually, not including suicides (5). Moreover, social crises such as the coronavirus disease 2019 (COVID-19) pandemic can also lead to an epidemic of depression. WHO estimated that the cases of the major depressive disorder increased by 53.2 million (27.6%) globally because of the COVID-19 pandemic in 2020 (6). As one of the most widespread diseases affecting human physical and mental health, depression is receiving increasing attention.

With the popularization of modern information and communication technology, time spent using screens on devices, such as mobile phones, laptops, tablets, computers, and televisions, is becoming a core component of daily life (7), online studying, or work. People’s use of display screen equipment (DSE) during their free time has substantially increased in recent years (8, 9). With the increased screen time, both sedentary behavior and exposure to radiofrequency electromagnetic fields (RF-EMF) increase (10). Poitras et al. (11) and Carson et al. (12) indicated that screen time is associated with unfavorable health outcomes, and Canada, Australia, and USA have developed the “Sedentary Behavior Guidelines” to provide guidance and motivate people to reduce their screen time (13–15). Several studies have indicated that increased screen time duration could be associated with lagged development (16), psychosocial symptoms (10, 17), obesity (18, 19), sleep disorders (20, 21), and cardiovascular disease (22, 23). Thus, active or passive screen use has become a common issue among both adolescents and adults.

The relationship between screen time and depression remains controversial, and the biological mechanisms underlying this possible association are unclear. Some studies have reported an independent and interactive relationship between screen time and physical activity (21, 23–26). Tremblay et al. (27) suggested that, independent of physical activity levels, screen time-based sedentary behaviors were associated with an increased risk of various physiological and psychological problems. Furthermore, previous studies indicated that RF-EMF exposure may increase the risk of headaches, fatigue, sleep problems, chronic tinnitus, and depression (10, 28). Liu et al. (7) and Wang et al. (29) conducted meta-analyses in 2015 and 2019, respectively, to investigate the relationship between screen time-based sedentary behavior and depression. Although screen time is the time spent using devices with display screens (30), some studies have combined DSE use with other behaviors requiring low energy expenditure, such as sitting, driving, and reading, when defining the total duration of sedentary behavior, which should also be given attention. Considering the influence of RF-EMF on psychological problems, we summarized the available evidence and performed a meta-analysis of longitudinal studies to investigate the effects of screen time on depression risk. To achieve our goal, we applied the patients, intervention, comparison, and outcome (PICO) framework as follows: human being (P), time spent on screen-based equipment (I), no or little time spent on screen-based equipment (C), and increased depression prevalence (O).

## 2 Methods

### 2.1 Literature search strategy

This meta-analysis was conducted following the checklist of the Meta-analysis of Observational Studies in Epidemiology (MOOSE) guidelines (31) and the Preferred Reporting Items for Systematic Review and Meta-analysis (PRISMA) statement (Supplementary File 1) (32). We systematically searched the PubMed, Embase, Web of Science, and China National Knowledge Infrastructure (CNKI) databases from their inception to May 2022 for studies describing an association between screen time and depression risk regardless of language or publication status. We used the following keywords: “screen time,” “video game,” “computer use,” “watching television,” “television view,” “internet use,” “electronic game,” “smartphone use,” “tablets,” or “iPads” in combination with “depression” or “depressive symptom” as search terms. In addition, all listed references were reviewed.

### 2.2 Inclusion and exclusion criteria

Studies were included if they fulfilled the following criteria: (1) the exposure of interest was screen time, (2) the outcome of interest was depression, (3) the study design was longitudinal, and (4) provided risk estimates such as hazard ratios (HR), relative risks (RR), or odds ratios (OR) with corresponding 95% confidence intervals (CIs) or sufficient data to calculate them. Studies were excluded if they were as follows: (1) publications that were not full reports, (2) duplicate studies, (3) studies on screen time addiction, (4) studies with inadequate information to calculate risk estimates, or (5) studies reporting time spent on other behaviors as well, causing difficulty in separating only screen time. Two reviewers (QZ and DY) independently reviewed all identified studies by title and abstract or full text. Disagreements were resolved through consultation with a third reviewer (CW).

### 2.3 Data extraction

The following information was extracted for each included study: first author’s name, publication year, study source, country, follow-up years, participants’ age range or mean age at the baseline, participants’ gender, sample size, screen equipment, depression measurement, depression definition, depression at baseline, study period, source of participates, screen time measurement, screen time in exposure group, screen time in the control group, depression at the baseline or adjusted, adjusted covariates, and effect estimates with their corresponding 95% CIs. Data extraction was conducted independently by two authors (JZG and QZ). Interobserver agreement was assessed using Cohen’s kappa (κ), and any disagreements were resolved by discussion with a third author (ZL).

### 2.4 Quality assessment

The methodological quality of the included studies was independently assessed by two reviewers (LL and GZ) using the Newcastle–Ottawa Scale (NOS) (33), which assesses the quality of cohort studies. The NOS includes eight items grouped into categories of selection, comparability, and outcome. Each study is assigned a score ranging between 0 and 9, and NOS scores over six indicate relatively high quality, five and six indicate medium quality, and less than five indicate low quality.

### 2.5 Statistical analysis

The RR was considered the common measure of the association between screen time and depression. The ORs were transformed into RRs using the formula $R\,\frac{O\,R}{\left(1-P_{0}\right)+\left(P_{0}\times O\,R\right)}$ where *P*_0_ indicates the incidence of the outcome of interest in the non-exposed group (34) and then synthesized with the RRs and the HRs into pooled RRs. The multivariable-adjusted RRs were preferentially pooled when such estimates were reported. If no adjusted analysis was available, the unadjusted estimate would be pooled. A fixed-effect model was applied when heterogeneity was not detected; otherwise, a random-effect model was used to summarize RRs for the association between screen time and depression. For further assessment of the association between screen time and depression risk, a subgroup analysis was conducted to explore sources of potential heterogeneity and examine the robustness of the primary results. This difference among subgroups was tested using meta-regression analysis (STATA “metareg” command). In sensitive analysis, we conducted a leave-one-out analysis to observe the magnitude of influence on the pooled RR of each study. Statistical heterogeneity among studies was evaluated using *I*^2^ statistics where values of 25, 50, and 75% represented the cutoff points for low, moderate, and high degrees of heterogeneity, respectively. Potential publication bias was evaluated using a funnel plot and Begg’s and Egger’s tests.

## 3 Results

### 3.1 Literature search

**FIGURE 1:**
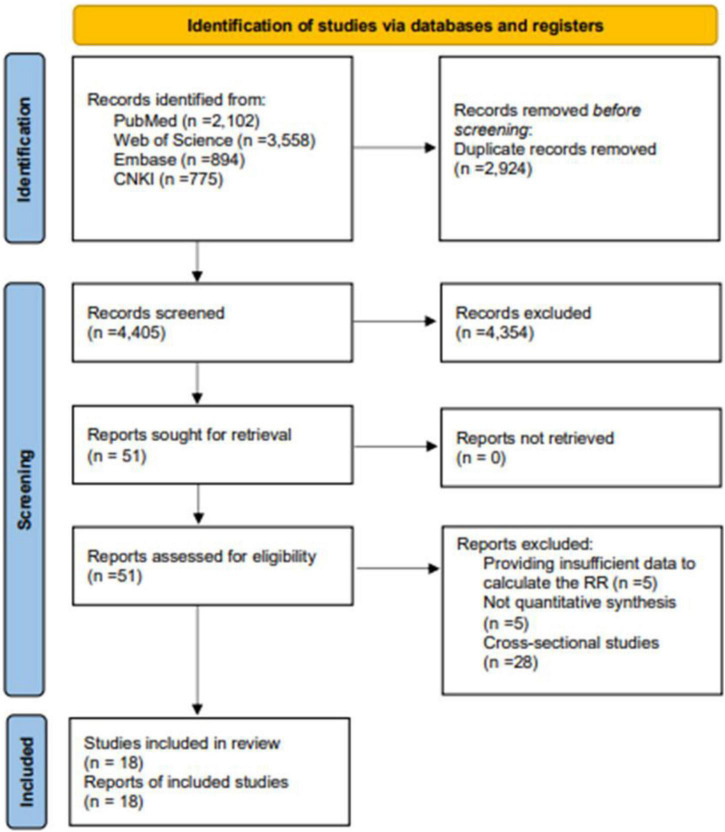
Flow chart of study selection.

Figure 1 shows all steps and reasons for study exclusion. The process of screening studies on databases retrieved 7,329 studies in total, of which 3,558 were from the Web of Science, 2,102 were from PubMed, 894 were from Embase, and 775 were from the CNKI. After eliminating reviews and duplicate publications, 2,924 articles were excluded. After screening studies by titles and abstracts, 51 remained. At the full-text review stage, 28 articles were excluded for having a cross-sectional design. Of the remaining 23 longitudinal studies, five provide insufficient data for calculating risk estimates. Ultimately, 18 studies were included in the meta-analysis.

### 3.2 Study characteristics

Table 1 and Supplementary File 2 show the characteristics of the 18 included studies (35–52). Overall, the studies included 241,398 individuals, with sample sizes ranging from 435 (51) to 100,517 (49). All included studies were observational studies. Half were conducted in Europe (35, 40, 44, 45, 47, 49–52), while five (38, 41, 42, 46, 48) and four (36, 37, 39, 43) were in Asia and North America, respectively. Two studies (43, 46) only included female participants, while four (36, 44, 45, 47) reported the results for male and female participants, respectively. Most of the included studies had been published since 2010, with only one study published in 2009 (41). The follow-up durations ranged from 2 (48, 52) to 144 months (51). Only one study (46) used a physician’s diagnosis or medication history as the assessment tool for depression, while the others used self-report questionnaires or scales. Eleven studies (35–38, 41, 44–46, 49–51) excluded baseline cases of depression, while seven (39, 40, 42, 43, 47, 48, 52) studies did not. In all included studies, screen time duration was based on self-report. Table 2 shows the results of the quality assessment. Based on NOS scores, 14 studies (35–39, 41–46, 49–51) were of moderate or high quality, and four (40, 47, 48, 52) were of weak quality.

**TABLE 1:** Characteristics of included studies in the meta-analysis.

First author	Study name	Country	Sample size	Age at baseline (years)	Gender	Study period	Screen equipment	Depression measurement	Depression at baseline	Follow-up time (months)	Covariates adjustment
Primack et al. (41)	Add Health	America	4,142	Mean 21.8 ± 1.82	M/F	General	TV, videocassettes, computer games	CES-D	Excluded	84	Sex, age, race, ethnicity, maternal educational level, the participants’ marital status and highest level of educational attainment at follow-up
Lucas et al. (46)	the Nurses’ Health Study	USA	49,821	Range 30–55	F	General	TV	Both depression diagnosis and use of antidepressants	Excluded	120	Age, time interval, current postmenopausal hormonal use, body mass index, marital status, involvement in a social or community group, smoking status, total energy intake, coffee intake, reported diagnosis of diabetes mellitus, cancer, myocardial infarction or angina, high blood pressure, rheumatoid arthritis, osteoarthritis, asthma, emphysema, categories of television watching, categories of physical activity, physical limitations in 1992, five-item Mental Health Index score in 1992
Thomée et al. (44)	None	Sweden	4,156	Range 20–24	M/F	General	Mobile phone	Two items from Prime-MD	Excluded	12	Relationship status, educational level, and occupation
Thomée et al. (45)	None	Sweden	4,163	Range 20–24	M/F	General	Computer	Two items from Prime-MD	Excluded	12	Relationship status, educational level, and occupation
Grøntved et al. (51)	EYHS	Danish	435	Mean 15.6 ± 0.4	M/F	General	TV and computer	MDI	Excluded	144	Age at baseline, follow-up time, sex, parental education level, parental marital status, smoking status, and alcohol intake in adolescence, and with school id treated as a random effect, BMI in adolescence, cardiorespiratory fitness in adolescence
Sui et al. (38)	ACLS	America	4,802	Range 18–80; mean 48.4 ± 9.8	M/F	General	TV	CES-D	Excluded	464	Age, gender, education, marital status, employment status, current smoker, body mass index, diabetes, and MVPA (hours/week)
Padmapriya et al. (43)	GUSTO	Asia	1,144	Mean 30.7 ± 5.1	F	During pregnancy	TV	EPDS	Included	17	Age, education, working during pregnancy, household income, smoking history, parity during pregnancy, and pregnancy BMI
Wu et al. (39)		China	2,521	Mean 18.43 ± 0.96	M/F	General	Video; computer, TV/video programs	CES-D	Included	14	Sex, age, residential background, BMI, perceived family economy, sleep quality, smoking, alcohol intake, exercise after school and physical activity
Khouja et al. (40)	the Avon Longitudinal Study of Parents and Children, a UK-based prospective cohort study	UK	1,869	16	M/F	General	TV, computer, texting	CIS-R	Included	24	Sex, maternal age, anxiety at age 15, maternal anxiety and depression, maternal education, parental socioeconomic position, also adjusted for child IQ, parental conflict, presence of the child’s father, number of people living in the child’s home, bullying and family TV use in early life, time spent alone (weekdays or weekends, as applicable)
Liu et al. (37)		China	3,396	Range 14–24; mean 18.3 ± 1.7	M/F	General	Mobile phone	BDI	Excluded	8	Age, sex, and other sociodemographics with significant associations with LTMPU at baseline; lifestyle practice and health conditions with significant associations with LTMPU at baseline
Zink et al. (42)	H&H	USA	2,525	Range 13–16; mean 14.6	M/F	General	TV, computer/ videogame	RCADS	Included	12	Demographic characteristics of sex, age, race, ethnicity, and highest parental education, SES, BMI percentile based on self-reported height and weight using the age- and sex-normed CDC standardized guideline, baseline Major Depressive Disorder, baseline computer/videogame use, baseline television viewing
Choi et al. (49)	Phenotypic and genomic data from over 100,000 UK Biobank participants	British	100,517	Range 18+	M/F	General	TV, computer	PHQ-9	Excluded	96	Participant characteristics (sex, age, assessment center), sociodemographic factors (socioeconomic deprivation, employment status, household income, completion of higher education, urbanicity, household size), and physical health factors (BMI and reported physical illness or disability)
Meyer et al. (48)	the COVID-19 and Well-being Study	USA	2,327	Range 18+	M/F	During COVID-19 pandemic	Screen not specific	BDI	Included	2	Age and sex, public health guidelines, time point (nine time points; weeks 0–8), and the interaction of time with each factor
Sarris et al. (35)	the UK Biobank	UK	31,343	Range 37–73; mean 56.7 ± 8.1	M/F	General	TV, computer	An item from PHQ-9	Excluded	120	Age, gender, ethnicity, social deprivation, education, and BMI
Ayuso-Mateos et al. (52)	the Edad con Salud project	Spain	1,103	Range 18+ mean 54.8 ± 16.4	M/F	During COVID-19 pandemic	Screen not specific	CIDI	Excluded	2	Age, sex, education level, whether the participant lived alone (both before and during the lockdown), whether the participant had co-habited/was co-habiting with a relative isolated by COVID-19, whether the participant had been/was concerned about a relative/friend infected by COVID-19, whether the participant had been infected with COVID-19 and its severity, whether the participant had enough quietness at home to get proper rest, whether the house-hold economic situation had worsened due to the COVID-19 emergency, whether the participant had been unemployed due to the COVID-19 emergency, time a day spent in front of screens during the lockdown (working and non-working), pre- and post-measure levels of physical activity
Kandola et al. (47)	the Millennium Cohort Study	UK	7,701	11	M/F	General	Video games, social media and leisure-time internet use	sMFQ	Included	36	Gender, socioeconomic position (household income), baseline emotional symptoms, self-reported maternal history of a depression or anxiety diagnosis, the self-reported experience of bullying, self-reported physical activity, and standardized, BMI
Liu et al. (36)	MABC	China	2,490	0	M/F	General	TV, electronic products (mobile phones, tablets, computers and others)	SDQ	Excluded	48	Age, gender, number of siblings, delivery model, birth weight, maximum educational level of parents, family income, passive smoking, outdoor activities
Pimenta et al. (50)	the SUN Project	Spanish	12,691	Mean 36.7 ± 11.5	M/F	General	TV, computer	DSM-IV	Excluded	21	Gender, marital status, years of university education, working hours, living status, hanging out with friends, smoking, physical activity, total energy intake, Mediterranean diet score, baseline self-perception of competitiveness, anxiety, and dependence levels, baseline BMI, use of tranquilizers or anxiolytics, insomnia, sleeping hours

USA, the United States; UK, the United Kingdom; F, female; M, male; Add Health, the National Longitudinal Survey of Adolescent Health; EYHS, the Danish cohorts of the European Youth Heart Study; ACLS, Aerobics Center Longitudinal Study; GUSTO, the growing up in Singapore toward healthy outcomes; H&H, the Happiness and Health study; MABC, the Ma’anshan Birth Cohort prospective cohort study; SUN, the “Seguimiento Universidad de Navarra” project; TV, television; CES-D, the Center for Epidemiological Studies Depression scale; Prime-MD, Primary care evaluation of mental disorders; MDI, the Major Depression Inventory; EPDS, the Edinburgh Postnatal Depression Scale; CIS-R, self-administered, computerized version of the revised Clinical Interview Schedule; BDI, the Beck Depression Inventory; RCADS, the Revised Children’s Anxiety and Depression Scale; PHQ-9, the Patient Health Questionnaire-9; CIDI, the Chinese version of the computerized Composite International Diagnostic Inventory; sMFQ, short Moods and Feelings Questionnaire; SDQ, the Strengths and Difficulties Questionnaire; DSM-IV, the Diagnostic and Statistical Manual of Mental Disorders; BMI, body mass index; SES, socioeconomic status; MVPA, moderate and vigorous physical activity; LTMPU, long-time mobile phone use; CDC, the Centers for Disease Control and Prevention; SES, socioeconomic status; COVID-19, coronavirus disease 2019; IQ, intelligence quotient.

**TABLE 2:** Quality assessment of included cohort studies.

Original studies	Selection				Comparability	Outcome			Total score
	(1) Representa-tiveness of the exposed cohort	(2) Selection of the non-exposed cohort	(3) Ascertainment of exposure	(4) Demonstration that outcome of interest was not present at start of study	(1) Comparability of cohorts on the basis of the design or analysis	(1) Assessment of outcome	(2) Was follow-up long enough for outcomes to occur	(3) Adequacy of follow up of cohorts	
Primack et al. (41)	1	1	0	1	2	0	1	1	7
Lucas et al. (46)	0	1	0	1	2	0	1	1	6
Thomée et al. (44)	1	1	0	1	1	0	0	1	5
Thomée et al. (45)	1	1	0	1	1	0	0	1	5
Grøntved et al. (51)	0	1	0	1	2	0	1	0	5
Sui et al. (38)	1	1	0	1	2	0	0	0	5
Padmapriya et al. (43)	1	1	0	0	2	0	0	1	5
Wu et al. (39)	1	1	0	0	2	0	0	1	5
Khouja et al. (40)	1	1	0	0	2	0	0	0	4
Liu et al. (26)	1	1	0	1	2	0	0	1	6
Zink et al. (42)	1	1	0	0	2	0	0	1	5
Choi et al. (49)	1	1	0	1	2	0	1	1	7
Meyer et al. (48)	0	1	0	0	2	0	0	1	4
Sarris et al. (35)	1	1	0	1	2	0	1	0	6
Ayuso-Mateos et al. (52)	1	1	0	0	2	0	0	0	4
Kandola et al. (47)	1	1	0	0	1	0	0	0	3
Liu et al. (36)	1	1	0	1	2	0	0	1	6
Pimenta et al. (50)	1	1	0	1	1	0	1	1	6

### 3.3 Quantitative synthesis

**FIGURE 2:**
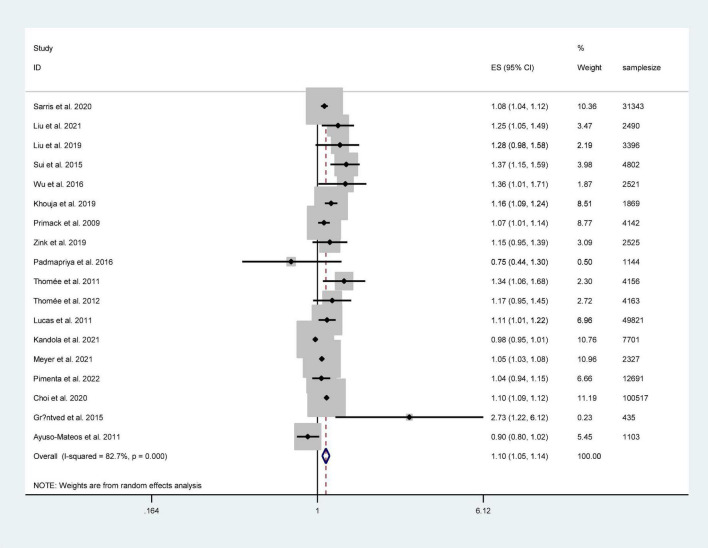
Forest plot of the association between screen time and the risk of depression.

Figure 2 shows the pooled results from the random-effect model. Among these studies, 12 reported a positive relationship between screen time and depression risk. The pooled RR was 1.10 (95% CI: 1.05–1.14). The results showed a positive association between screen time and depression risk with a high level of heterogeneity (*I*^2^ = 82.7%, *P* < 0.001).

### 3.4 Subgroup analysis

Subgroup analyses were conducted by study location, age, gender, sample size, study quality, depression at baseline, depression definition, follow-up time, screen equipment, whether during the COVID-19 pandemic, screen time in the control group, and physical activity controlled for or absent in the models (Table 3). When stratified by gender, the results showed a positive association between screen time and depression in female participants (RR = 1.15, 95% CI: 1.01–1.31, *I*^2^ = 76.2%); however, this association was not statistically significant in male participants (RR = 1.12, 95% CI: 0.89–1.42, *I*^2^ = 77.4%). Screen time was significantly associated with an increased risk of depression in participants who watched television (RR = 1.13, 95% CI: 1.06–1.21, *I*^2^ = 65.0%) and used mobile phones (RR = 1.13, 95% CI: 1.01–1.25, *I*^2^ = 57.7%). A significant association between screen time and increased depression risk was observed among participants from Asia (RR = 1.24, 95% CI: 1.07–1.43, *I*^2^ = 21.7%). The pooled results of studies that excluded baseline depression in their analyses suggested a significantly positive association between screen time and depression risk (RR = 1.11, 95% CI: 1.06–1.16, *I*^2^ = 66.0%).

**TABLE 3:** Subgroup analysis of odd ratios for the association between screen time and depression.

		No of studies	RR (95% CI)	*I*^2^ (%)	*P*-value for heterogeneity	*P*-value between groups
Study location						0.648
	Asia	4	1.24 (1.07, 1.43)	21.7	0.280	
	North America	9	1.07 (1.01, 1.14)	89.4	<0.001	
	Europe	5	1.11 (1.04, 1.18)	67.7	0.015	
During COVID-19 pandemic						0.101
	Not	16	1.12 (1.07, 1.18)	82.6	<0.001	
	Yes	2	0.98 (0.85, 1.14)	83.2	0.015	
Depression at baseline						0.530
	Excluded	12	1.11 (1.06, 1.16)	66.0	0.001	
	Included	6	1.07 (1.00, 1.15)	85.4	<0.001	
Sample size						0.221
	<5,000	13	1.14 (1.07, 1.22)	73.4	<0.001	
	>5,000	5	1.06 (1.00, 1.13)	92.5	<0.001	
Study quality						0.287
	Low quality	4	1.03 (0.96, 1.10)	90.6	<0.001	
	Medium quality	12	1.17 (1.09, 1.25)	56.8	0.008	
	High quality	2	1.10 (1.06, 1.12)	0	0.397	
Follow-up time						0.580
	<60 months	12	1.09 (1.03, 1.15)	79.4	<0.001	
	>60 months	6	1.11 (1.06, 1.16)	64.2	0.016	
Screen time in the control group						0.037
	Continuous	4	1.05 (1.01, 1.09)	64.8	0.036	
	≤1 h/day	7	1.12 (1.06, 1.18)	50.3	0.060	
	>1 h/day	4	1.22 (1.09, 1.36)	0	0.722	
**Physical activity adjusted**						
	Yes	8	1.11 (1.01, 1.22)	83.4	0.001	0.774
	No	10	1.10 (1.06, 1.14)	68.3	<0.001	
Sex						0.867
	Mix	12	1.10 (1.06, 1.14)	74.6	<0.001	
	Female	7	1.15 (1.01, 1.31)	76.2	0.001	
	Male	5	1.12 (0.89, 1.42)	77.4	0.004	
Gender						0.429
	0–20	7	1.18 (1.04, 1.34)	86.0	<0.001	
	20–44	8	1.08 (1.05, 1.12)	61.0	0.012	
	>44	3	1.09 (0.92, 1.30)	88.4	<0.001	
Screen equipment						0.689
	Screen not specific	6	1.06 (1.00, 1.13)	70.5	0.005	
	Using mobile phone	4	1.13 (1.01, 1.25)	57.7	0.069	
	Watching TV	8	1.13 (1.06, 1.21)	65.0	0.006	
	Using computer	8	1.09 (0.99, 1.20)	89.1	<0.001	

### 3.5 Sensitivity analysis

Sensitivity analysis was adopted to identify the potential sources of heterogeneity in the association between screen time and depression risk. This helped us to examine the influence of various exclusions on the combined RRs and test the stability of the quantitative synthesis results. The pooled RRs ranged from 1.09 (95% CI: 1.05–1.14) to 1.11 (95% CI: 1.06–1.16) when one study was omitted. The leave-one-out analysis indicated that none of the individual studies significantly influenced the overall results.

### 3.6 Publication bias

**FIGURE 3:**
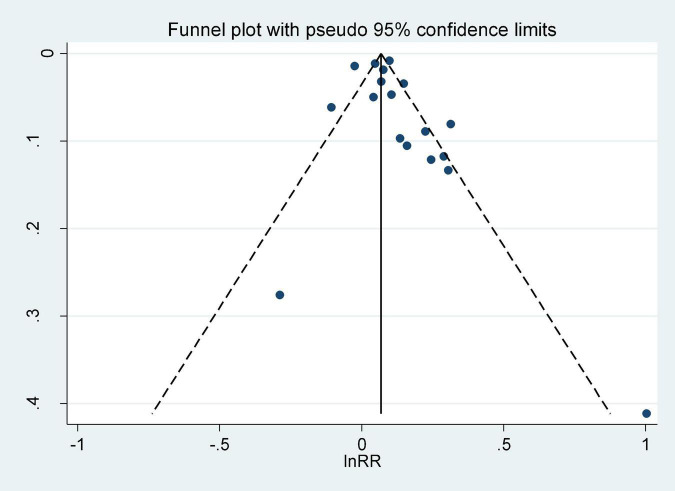
Funnel plot for studies of screen time in relation to the risk of depression.

Visual inspection of the funnel plot did not reveal any significant asymmetry (Figure 3). Egger’s and Begg’s tests showed no obvious publication bias across studies (Egger’s test *t* = 1.10, *P* = 0.272; Begg’s test *z* = 1.08, *P* = 0.296).

## 4 Discussion

We conducted a meta-analysis of 18 longitudinal studies to investigate the association between screen time and depression risk. The pooled results showed that screen time was associated with an increased risk of depression with an RR value of 1.10 (95% CI: 1.05–1.14; *I*^2^ = 82.7%).

All included studies were observational, with few conducting experimental validations, and the research value of observational studies will be affected if they are not rigorously designed. Experimental studies may provide an important proof of concept for intervention efficacy; however, they can sometimes be limited by factors, such as ethical or experimental conditions (53, 54). Although high heterogeneity was observed in the meta-analysis, subgroup and meta-regression analyses showed that study location, whether depression was excluded at baseline, screen time in the control group, and study quality could explain the potential heterogeneity. Heterogeneity moderately decreased when grouped by age and screen time category; thus, in addition to screen time duration, future studies should investigate the influence of screen time on different age groups and the effects of different screen mediums on mental health. Heterogeneity declined significantly as study quality improved. When studies that did not exclude depression at baseline were excluded, heterogeneity decreased to 54%. The original study’s design had a significant impact on the results, and future studies should consider study design rigor. In the subgroup analysis by screen equipment, watching television and using mobile phones increased the incidence of depression.

In the subgroup based on age, the association between screen time and depression was not statistically significant in the elders. Wang et al. (55) found that the elders who reported internet use had lower depression levels compared with those who did not. Another study on different types of internet use and depressive symptoms indicated that using the internet for social contact and entertainment decreased depression scores in the elderly, but when using the internet for learning, working, or commercial activity, the effect of relieving depressive symptoms disappeared (56). Elders spend more time online for recreational and leisure activities (56, 57). However, one study of internet use and mental health suggested that internet use affects mental health and increases the incidence of depressive symptoms in elders who may even experience feelings of technological panic (58). Future research should consider technological fear as an important influencing factor.

In the subgroup analysis by gender, screen time was significantly associated with an increased risk of depression in women; however, this association was not statistically significant in men. In general, screen time is considered a sedentary behavior, and this finding was consistent with previous meta-analyses on the association between sedentary behavior and mental health and screen time-based sedentary behavior on depression (29, 59). One explanation might be that women were more likely to be influenced by intimate social relationships that contribute to depression and thus would experience greater depressive symptoms than men. Kawachi et al. (60), Wirback et al. (61), and Altemus et al. (62) found that gender differences in support derived from social network participation may partly account for the higher prevalence of psychological distress among women compared with men, and smaller social networks, fewer close relationships, and lower perceived adequacy of social support have been linked to depressive symptoms (60–62). When screen time increases, women may have less time to communicate with others, which could result in developing fewer intimate relationships and smaller social networks, ultimately leading to mental disorders, such as depression.

Subgroup stratification according to whether a study was conducted during the COVID-19 pandemic showed that while screen time increased the risk of depression during the normal period, the opposite association was found during the COVID-19 pandemic (52). This may be because watching television and using mobile phones or computers could relieve an individual’s negative mood during lockdown conditions. Most of the subgroup effects were statistically significant. The population from Asia was at a higher risk than those from North America or Europe. An investigation of sitting time trends in 27 countries found that time spent on sedentary behaviors may not be increasing in the European region (8), and among these countries, the prevalence of depression has decreased steadily.

Electronic devices have become an integral part of many people’s lives, even among retired adults who do not need to work or study. In our study, we observed a significant effect of screen time on depression in youth (aged 0–20 years), although heterogeneity was high. Our findings support limiting screen time for adolescents. The results regarding the effect of screen time on depression in the elderly were not statistically significant, and our findings did not confirm if the relationship was positive or negative. Insufficient information in the original studies and the wide age range of participants made it difficult to perform a more detailed age group study. Future studies should focus on the effect of age and analyze the effect of screen time on depression risk separately for different age groups. Notably, the results stratified by subgroup, whether the study was conducted during the COVID-19 pandemic, suggested that screen time may alleviate depressive symptoms during lockdowns. Thus, the government should encourage people to use electronic devices to distract themselves and relieve their negative emotions during lockdown situations.

The underlying mechanisms of the relationship between screen time and depression risk remain unclear. With the increasing prevalence of depression, depression prevention has become a widespread public health concern. Therefore, identifying modifiable risk factors to aid in depression prevention is an important task. Several hypotheses may explain the impact of screen time on depression. First, increased screen time led to curtailed physical activity, which has been beneficial for reducing depression risk (63, 64). However, when stratified by physical activity regardless of if it was adjusted, the pooled results still indicated that screen time was a risk factor for depression. Tremblay et al. (27) suggested that independent of physical activity levels, screen time-based sedentary behaviors are associated with increased depression risk. As sedentary behavior and physical activity are both common human experiences, future studies are needed to explore the relationship between both behaviors and depression. Second, screen time, such as watching television and using mobile phones or computers, has been associated with sleep disorders, which could lead to sleep problems and increase the risk of depression (46, 65). Third, according to the causal model of social networks and social supports, social ties have protective effects on mental health and direct communication helps individuals build intimacy (60). Screen time can cause a reduction in social interactions and narrow social networks, and a lack of social networks may lead to social solitude and lower perceived adequacy of social support; thus, such changes could result in depressive symptoms. Fourth, RF-EMF may provide another explanation. With the rapid development and widespread use of electronic devices, health authorities have recognized the possible effects of long-term exposure to RF-EMF. Recent studies reported that RF-EMF may be linked to adverse health consequences, and several countries have made proposals to reduce the use of electronic devices. However, research findings on the relationship between EMFs and health problems have been inconsistent. Therefore, future studies should consider potential confounding or interactive effects, explore the underlying biological mechanism, and demonstrate an independent effect of screen time on depression risk.

### 4.1 Strengths and limitations

This meta-analysis highlights the synthesized effects of screen time on depression risk. First, our research was based on cohort studies, which provided much stronger and more sufficient evidence. Second, it included a total of 237,146 participants from seven countries across Europe, Asia, and North America. The large sample size and wide range of locations significantly increased the statistical power and generalizability of the findings on the association between screen time and depression risk. Third, no obvious publication bias was detected in our study, which indicates that the combined results are reliable and convincing overall. Finally, our study excluded mixed sedentary behavior and screen time that could not be separated.

Some of this study’s potential limitations should also be discussed. First, recall bias and measurement errors are unavoidable when using self-report questionnaires to assess screen time. Electronic devices, such as mobile phones, computers, and smart televisions, can record time spent using the device in detail, thereby allowing for screen time to be measured objectively and conveniently. Researchers should obtain as much detailed information as possible and use objective measurements in future studies. Second, although included studies had adjusted for confounding factors, some of the included studies did not adjust for potential confounders, such as age, gender, and physical activity level, which might influence the association between screen time and depression risk. Future studies should pay more attention to these important covariates and measure and adjust for key variables. Third, the information in the included original studies was limited; therefore, we were unable to conduct dose-response analyses. Objective measurements of screen time are recommended, so we can easily collect more definite and detailed information about exposure to screen time and further analyze the dose-response effects.

## 5 Conclusion

This meta-analysis demonstrates that screen time is likely to increase the risk of depression. The high heterogeneity may be the result of a less rigorous original study design across the included studies. The effects of screen time on depression risk may vary widely, and the determinants or the benefits of screen time were shown to differ based on duration and individual characteristics (e.g., age, gender, and location). Among young and female populations, screen time was found to significantly increase depression risk. Compared with less screen time, screen time exceeding 1 h/day was associated with a higher risk of depression. Our findings support the recommendations to limit the prolonged use of electronic devices. Objective measures are recommended to use in future studies to explore complex relationships and specific time constraints of screen time. Considering the increasing prevalence of depression in modern society and the increasing number of people exposed to screens for long periods, our findings have important implications for depression prevention.

## Data availability statement

The raw data supporting the conclusions of this article will be made available by the authors, without undue reservation.

## Author contributions

CW and LL conceived the study. GZ, HH, JZ, XK, SY, DY, and ZC were responsible for the collection and cleaning of the data and assisted in the writing of the manuscript. LL and QZ wrote the manuscript. LZ, YG, and ZL contributed to the review and revision of the study. CW was the guarantor of this work and, as such, had full access to all the data in the study and takes responsibility for the integrity of the data and the accuracy of the data analysis. All authors contributed to the article and approved the submitted version.

## Supplementary material

Supplementary File 1 The PRISMA checklist.

Supplementary File 2 Details of included articles.
